# Supplementary material for: The Role of Polycystic Kidney Disease-Like Homologs in Planarian Nervous System Regeneration and Function
Source: Integr Org Biol. 2024 Sep 10;6(1):obae035. doi: 10.1093/iob/obae035 (PMC11448475; doi:10.1093/iob/obae035)

Supplementary Information

The role of *polycystic kidney disease-like* homologs in planarian nervous system regeneration and function

Kelly G. Ross, Sarai Alvarez Zepeda, Mohammad A. Auwal, Audrey K. Garces, Sydney Roman, and Ricardo M. Zayas

Figure S1. A Bayesian inference phylogeny of PKD1 and PKD1L proteins. Node support values are listed as percent support next to the relevant node.

Figure S2. A Bayesian inference phylogeny of PKD2 proteins. Node support values are listed as percent support next to the relevant node.

Figure S3. Timelines for RNAi experiments and vibration assay. (A) Timeline for *pkd* RNAi experiments and subsequent behavioral testing. All testing was performed between 13- and 15-days following amputation. F, feed; D, day in an experiment; DPA, days post amputation. (C-E) depictions of the mechanosensory assays. (C) An illustration of the tapping device used to test mechanosensation is in Figure 4 (C’). A photo of the tapping device setup is shown. (D-E) Illustrations and still images from behavioral videos showing how pre-stimulus “gliding” length and post-stimulus “contraction” length were measured for the rheosensory (D) and vibration (E) assays. The worms depicted are examples of a typical wild-type behavioral response. (F-G) Worms with *pkd* knockdowns that demonstrated a significant loss of mechanosensory function in the vibration assay were also tested using the rheosensory assay to validate

the observed loss of mechanosensation in intact (F) and regenerate (G) worms. The percent change in length was calculated and plotted in a box plot wherein the box extends from the 25th to 75th percentiles of the range, and the bisecting line represents the median. The whiskers extend the full range of the data. *pkd1L-3*, which did not display a mechanosensory phenotype, was included as an additional negative control. n = 8-13 worms for each experimental group. ****$p < 0.0001$; all other groups are non-significant ($p > 0.01$); One-way ANOVA with Dunnett's multiple comparisons test.

Figure S4. Chemosensation assay. (A) Illustration of the custom-made chamber used for chemosensation assays displaying the markings on the dish used to determine inclusion in the 'feeding zone' and start zone as explained in the methods. The lines were drawn on the bottom of the clear dish and could be visualized in the videos, which were taken with an overhead camera. (A') Photo of trays with drawn lines used in this study.

Table S1. Results of the PKD domain BLAST searches against the planarian transcriptome.

Table S2. The primers and eBlock sequences that were used in this study.

File S1. Protein sequences used for the PKD1 phylogenetic analysis tree shown in Figure S1.

File S2. Protein sequences used for the PKD2 phylogenetic analysis tree shown in Figure S1.

File S3. PKD1 cation channel alignment used for the phylogenetic analysis tree shown in Figure S1.

File S4. PKD2 cation channel alignment used for the phylogenetic analysis tree shown in Figure S2.

File S5. Link to Arduino code for controlling tapping device with servo motor

Video S1. Control worms 28 days following the first RNAi feeding display normal gliding locomotion movements in the arena used for the chemosensory assay.

Video S2. *pkd2-4(RNAi)* worms 28 days following the first RNAi feeding display normal gliding locomotion movements in the arena used for the chemosensory assay.

Video S3. Control worms at 55 days following the first RNAi feeding display normal gliding locomotion movements.

Video S4. *pkd2-4(RNAi)* worms 55 days following the first RNAi feeding display slow, jerky locomotion movements.

Supplementary Figure 1

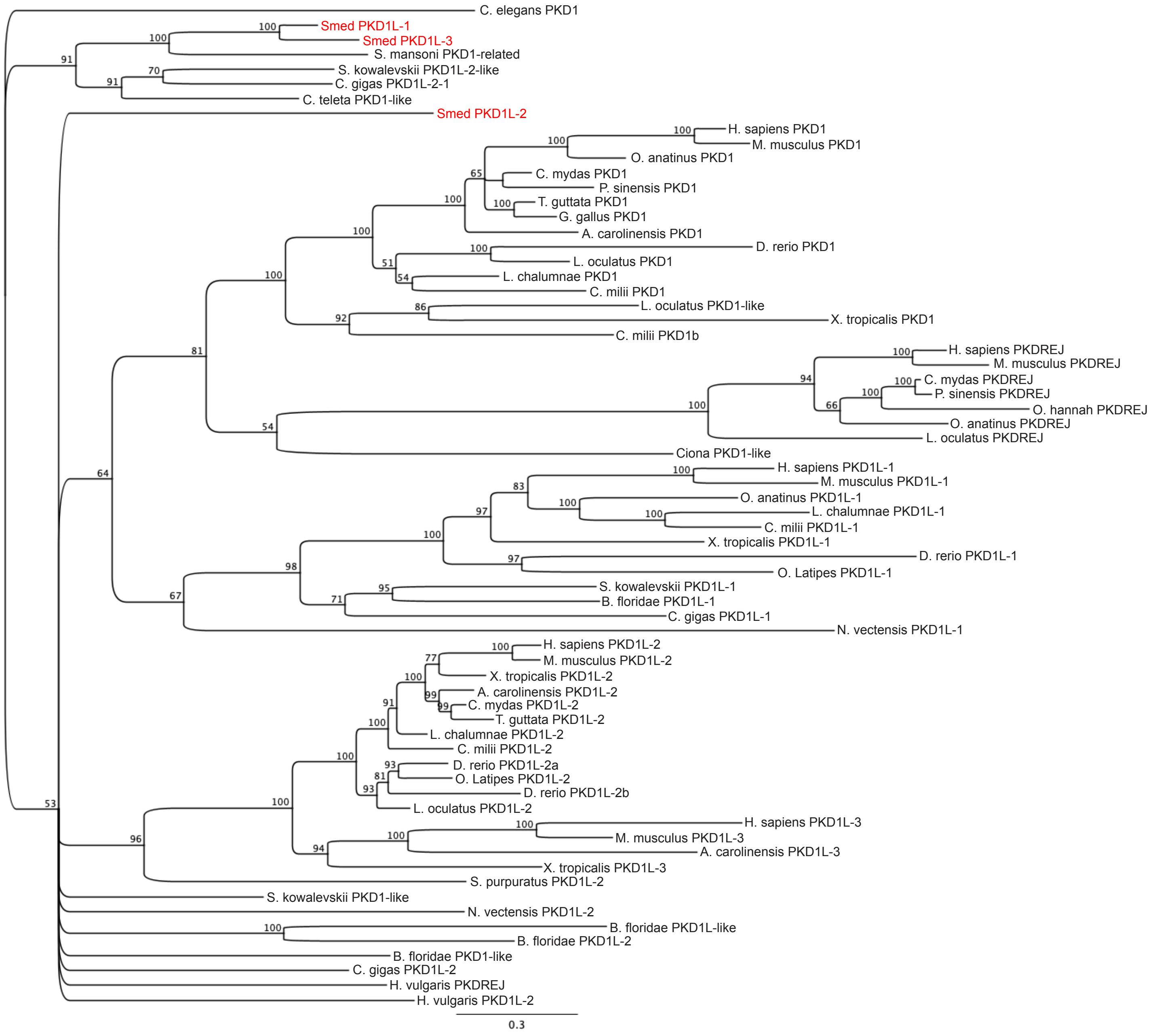

Supplementary Figure 2

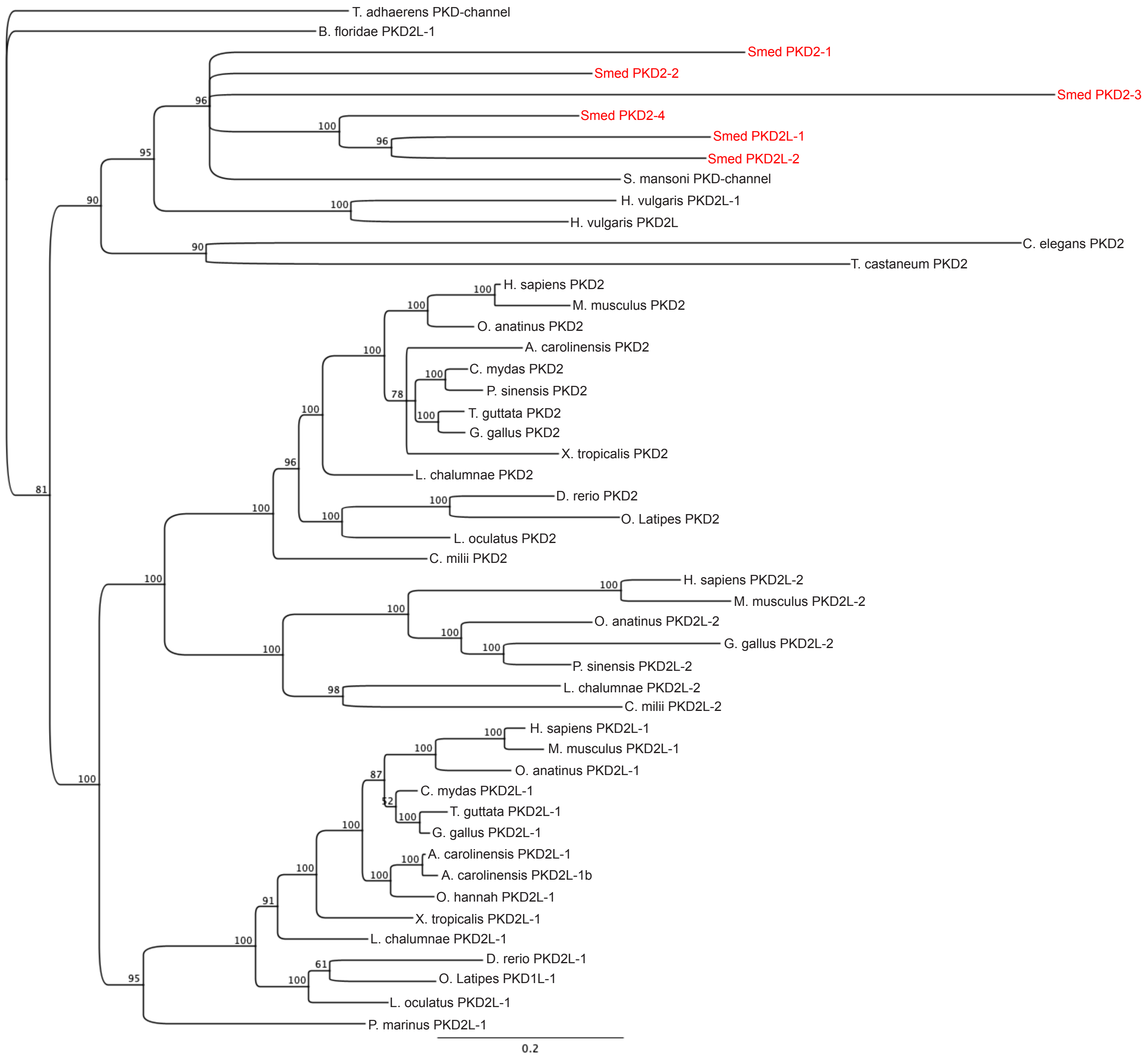

Supplementary Figure 3

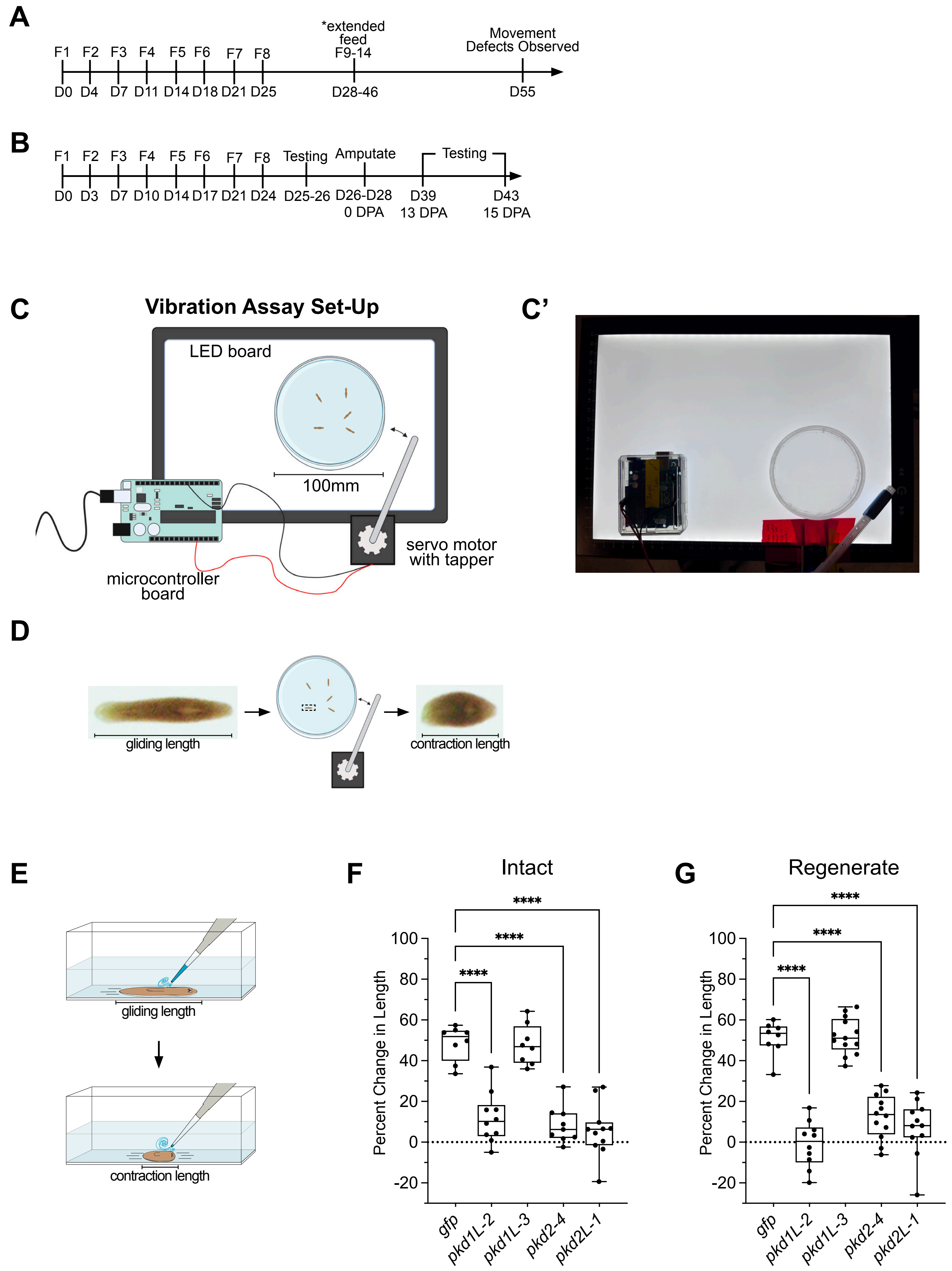

Supplementary Figure 4

Chemosensation Assay Set-Up

A

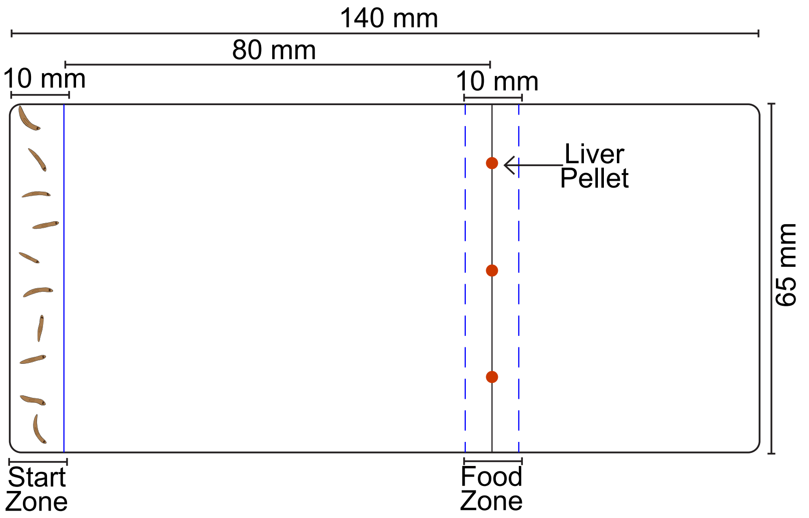

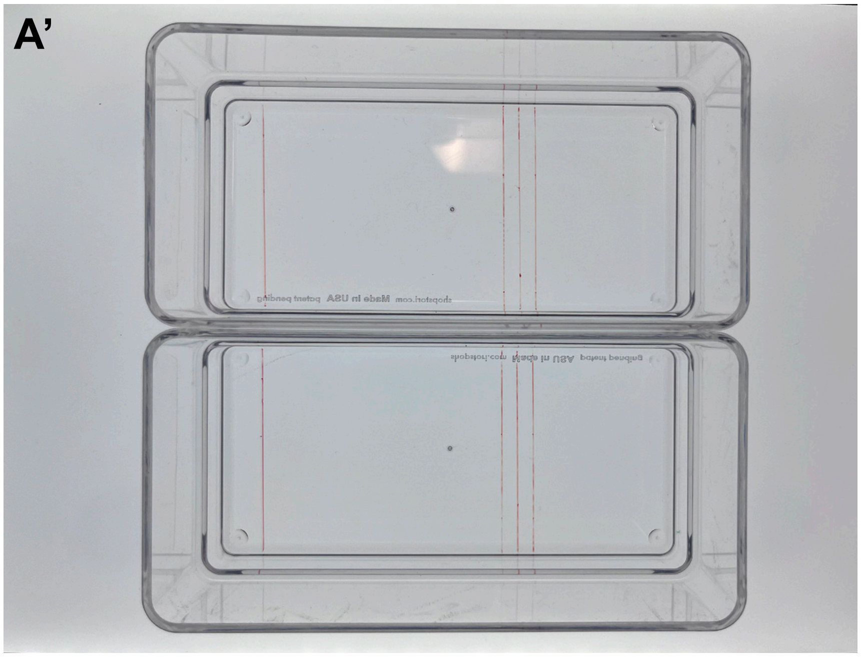

Supplement: obae035_Supplemental_Files [file obae035_supplemental_files.zip › FINAL IOB-2024-021.R1 Supplmentary Information and Figures.pdf]
